# Inhibition of Cx43 mediates protective effects on hypoxic/reoxygenated human neuroblastoma cells

**DOI:** 10.1111/jcmm.13177

**Published:** 2017-05-09

**Authors:** Nunzio Vicario, Giovanna Calabrese, Agata Zappalà, Carmela Parenti, Stefano Forte, Adriana Carol Eleonora Graziano, Luca Vanella, Rosalia Pellitteri, Venera Cardile, Rosalba Parenti

**Affiliations:** ^1^ Department of Biomedical and Biotechnological Sciences Physiology Section University of Catania Catania Italy; ^2^ Department of Drug Sciences University of Catania Catania Italy; ^3^ IOM Ricerca Viagrande Italy; ^4^ Institute Neurological Sciences National Research Council Catania Italy

**Keywords:** olfactory glia, growth factors, neuroprotection, gap junctions, connexin 43

## Abstract

Olfactory ensheathing cells (OECs), a special population of glial cells, are able to synthesise several trophic factors exerting a neuroprotective action and promoting growth and functional recovery in both *in vitro* and *in vivo* models. In the present work, we investigated the neuroprotective effects of OEC‐conditioned medium (OEC‐CM) on two different human neuron‐like cell lines, SH‐SY5Y and SK‐N‐SH (neuroblastoma cell lines), under normoxic and hypoxic conditions. In addition, we also focused our attention on the role of connexins (Cxs) in the neuroprotective processes. Our results confirmed OEC‐CM mediated neuroprotection as shown by cell adherence, proliferation and cellular viability analyses. Reduced connexin 43 (Cx43) levels in OEC‐CM compared to unconditioned cells in hypoxic conditions prompted us to investigate the role of Cx43‐Gap junctions (GJs) and Cx43‐hemichannels (HCs) in hypoxic/reoxygenation injury using carbenoxolone (non‐selective GJ inhibitor), ioxynil octanoato (selective Cx43‐GJ inhibitor) and Gap19 (selective Cx43‐HC inhibitor). We found that Cx43‐GJ and Cx43‐HC inhibitors are able to protect SH‐SY5Y and allow to these cultures to overcome the injury. Our findings support the hypothesis that both OEC‐CM and the inhibition of Cx43‐GJs and Cx43‐HCs offer a neuroprotective effect by reducing Cx43‐mediated cell‐to‐cell and cell‐to‐extracellular environment communications.

## Introduction

The olfactory system is a specific area of the central nervous system (CNS) capable of supporting neurogenesis throughout the life of mammals by forming new olfactory receptor neurons (ORNs) 1, 2, 3. These neurons are then able to spread axons from the peripheral nervous system of the olfactory epithelium into the CNS environment of the olfactory bulb 4. The capacity of ORNs to stimulate neurogenesis in the adult olfactory system may be due to both the neural stem cells present in the olfactory epithelium and the glial cells known as olfactory ensheathing cells (OECs) 5, 6. Olfactory ensheathing cells, originally described by Golgi and Blanes 7, 8, 9 at the end of the 19th century, are a special glial cell population sharing properties with both Schwann cells and the astrocytes 10, 11. Similarly to Schwann cells, OECs express some characteristic markers such as the low affinity neurotrophin receptor (p75NTR) and adhesion molecules such as laminin, L1 and N‐CAM; likewise to astrocytes they express the S‐100 protein and the glial fibrillary acidic protein (GFAP), a member of the intermediate filament family that offers support to glial cells 12, 13.

Furthermore, OECs are able to secrete high level of growth factors, such as nerve growth factor (NGF), basic fibroblast growth factor (bFGF), brain derived neurotrophic factor (BDNF), glial derived neurotrophic factor (GDNF), ciliary neurotrophic factor (CNTF), neurotrophins NT4, NT5 and neuregulins, which exhibit important functions as neuronal supporting elements 14, 15, 16, 17, 18, 19.

Gap junctions (GJs) are specialized intercellular channels that directly connect cytoplasms of adjacent cells, enabling direct exchanges of small molecules (less than 1200 Da). Gap junctions are composed by two docked hemichannels (HCs), also named connexons, one on each cell. Hemichannels are hexamers of homotypic or heterotypic connexins (Cxs), which are the transmembrane proteins encoded by a multigene family of approximately 20 members in mammals 20, 21, 22, 23, 24, 25 that form respectively homotypic or heterotypic GJs.

Besides constituting ‘GJs plaques’ that allow GJ intercellular communication (GJIC), Cxs also constitute free HCs throughout the plasma membrane, allowing exchange of a number of autocrine and paracrine signalling molecules between the cytoplasm and the extracellular environments 26, 27, 28.

Gap junctions, as well as HCs, play a crucial role in a wide range of cellular activities, including cell signalling, differentiation, growth, pro‐apoptotic signalling, either as anti‐apoptotic gates or pathogenic pores depending on conditions and cell type 29, 30, 31, 32. Gap junctions and free HCs are extensively distributed in different tissues and organs 33 in which different cell type usually expresses specific Cxs profile involved in the selective permeability of channels formed, according to the metabolic or functional needs 34, 35, 36, 37, 38. Also in the CNS, GJs and HCs are extensively distributed among neurons and glial cells 39, 40, 41, where, contributing to GJIC and cell‐extracellular communication, they provide specific exchange pathways under resting conditions, and also play a context‐dependent role in contradictory cell survival or cell death phenomena 42.

In recent years, much attention has been paid to the exploitation of neuroprotective effects in combatting the progression and chronicity of neurodegeneration. In particular, evidence shows altered Cxs expression and functions under pathological conditions, suggesting a central role of glial GJs and HCs in the development of various neurodegenerative diseases 21, 43, 44, 45, 46. Conversely, the therapeutic potential of OECs is attracting considerable interest owing to their exceptional ability to promote functional recovery of the damaged CNS 3, 47, 48, 49, 50. However, the molecular mechanisms underlying this protective function are not yet known. The aim of this study was firstly to investigate the neuroprotective effects of OEC conditioned medium (OEC‐CM) on two different neuroblastoma cell lines (SH‐SY5Y and SK‐N‐SH) exposed to hypoxic/reoxygenation (H/R) injury. Towards this goal we explored the relationship between OEC‐CM and Cxs, HCs and GJs following H/R injury in cultures grown with and without OEC‐CM. We found that: (1) OEC‐CM offers a neuroprotective effect to the SH‐SY5Y and SK‐N‐SH cells exposed to H/R injury; (2) injured SH‐SY5Y and SK‐N‐SH cells show higher Cx43 levels compared to normoxic cultures; and (3) H/R cultures grown in the presence of GJ and/or HC chemical inhibitors, such as carbenoxolone (CBX, non‐selective GJIC inhibitor) 51, ioxynil octanoato (IO, Cx43 homotypic GJ inhibitor) 52, and Gap19 (Cx43 homotypic HC inhibitor) 53, show higher viability compared to control cultures. Our results, showing the neuroprotective effects of OEC‐CM likely afforded by inhibition of Cx43 GJ/HC signalling pathways, suggest potential new therapeutic tools against neurodegenerative diseases.

## Materials and Methods

### Primary olfactory ensheathing cell cultures

Primary OECs were isolated from post‐natal day 0 (P0) CD1 mice olfactory bulbs. Experiments were performed in compliance with current guidelines for animal care and in accordance with the European Community Council Directive (86/609/EEC). All efforts were made to minimize animal suffering and to use the fewest animals possible.

Ten P0 pups were decapitated, the entire brain was exposed and bulbs removed and dissected out in cold (+4°C) Leibowitz L‐15 medium (Sigma‐Aldrich, Saint Louis, Missouri, USA). Subsequently, collected olfactory bulbs were digested twice in fresh minimum essential medium‐H (MEM‐H; Sigma‐Aldrich, Saint Louis, Missouri, USA) containing 0.03% collagenase (Sigma‐Aldrich, Saint Louis, Missouri, USA) and 0.25% trypsin (Sigma‐Aldrich, Saint Louis, Missouri, USA) for 15 min at 37°C. Suspension was mechanically triturated and filtrated through a 80 μm nylon filter and centrifuged at 600 g for 10 min. Cells were suspended with fresh complete Dulbecco's modified Eagle's medium (DMEM, Sigma‐Aldrich, Saint Louis, Missouri, USA) supplemented with 10% foetal bovine serum (FBS, Sigma‐Aldrich, Saint Louis, Missouri, USA), 2 mM L‐glutamine (Sigma‐Aldrich, Saint Louis, Missouri, USA), penicillin (50 U/ml, Sigma‐Aldrich, Saint Louis, Missouri, USA) and streptomycin (50 mg/ml, Sigma‐Aldrich, Saint Louis, Missouri, USA) and plated in 25 cm^2^ flasks. Cytosine arabinoside (Sigma‐Aldrich, Saint Louis, Missouri, USA) at final concentration of 10^−5^ M was added 24 hours (hrs) after plating to reduce the number of dividing fibroblasts. After two passages, purity of OECs was verified by using immunofluorescence with p75 and S‐100 (data not shown). Media were replaced twice a week for three passages and then cultures were used to collect the conditioned medium. OEC‐CM was removed from the cultures and filtered through a membrane filter (0.22 μm pore diameter) to remove cells and debris.

### Neuroblastoma cell lines cultures

The human neuroblastoma cell lines, SH‐SY5Y and SK‐N‐SH, were purchased from ATCC (Rockville, MD, USA) and grown as previously described 54. Briefly, cells were incubated at 37°C in a humidified 5% CO_2_/95% air atmosphere and maintained in DMEM/F12 (Sigma‐Aldrich) supplemented with 10% FBS (Sigma‐Aldrich), 2 mM L‐glutamine (Sigma‐Aldrich), penicillin (50 U/ml, Sigma‐Aldrich) and streptomycin (50 mg/ml, Sigma‐Aldrich, Saint Louis, Missouri, USA). The medium was replaced twice a week.

### Hypoxic/reoxygenation injury and OEC‐CM treatment

Cells at a density of 2.5 × 10^4^ cells/cm^2^ were seeded on appropriate supports for analysis 16 hrs before performing the experiments (time 0). The following experimental culture groups were established: untreated control groups (CTRL Normoxia) and hypoxic groups (CTRL Hypoxia) at 0, 3, 8 and 24 hrs. Each group was grown in normal culture conditions for 16 hr, after which they were subjected to hypoxia (1% O_2_) for 3 hrs, followed by reoxygenation to 24 hrs. All cultures were grown with DMEM‐F12 (89%), FBS (10%) and P/S (1%).

For OEC‐CM treatment, at time 0, media conditioning (25%, 50% and 100%) obtained from OECs cultured for 24, 48 and 72 hrs were applied in the same experimental culture groups. To verify the status of hypoxia, the expression of hypoxia‐inducible factor 1‐alpha (HIF1A), a specific transcription factor of the hypoxic response, was analysed by immunofluorescence (data not shown).

### Immunofluorescence

Immunofluorescence analysis on SH‐SY5Y cells was performed as previously described 54. Briefly, after fixation with 4% Paraformaldehyde (PFA), cells were permeabilized in 0.2% Triton X100 and blocked by incubation with 10% normal goat serum (NGS, Gibco, Invitrogen, Milan, Italy) for 1 hr at room temperature (RT). The primary incubation was performed, overnight at 4°C, with the following antibodies: rabbit anti‐p75 (1:500, Chemicon Int. Inc., USA), mouse anti‐S‐100 (1:100; Sigma‐Aldrich, Saint Louis, Missouri, USA), rabbit anti‐HIF1A (1:200; Sigma‐Aldrich, Saint Louis, Missouri, USA), mouse anti‐Cx43 (1:150, Cell Signaling, Danvers, Massachusetts, USA) and rabbit β‐tubulin (1:200, Cell Signaling, Danvers, Massachusetts, USA).

After washing, slides were incubated with the appropriate secondary antibodies: fluorescence isothiocyanate (FITC) labelled anti‐rabbit antibody (1:200, Chemicon, Int. Inc., USA) and Cy3 labelled antimouse antibody (1:1000 Chemicon, Int. Inc., USA) for 1 hr at RT. Nuclei were stained with DAPI (1:1000) for 5 min. Finally, slides were mounted in fluorescent mounting medium Permafluor (Thermo Scientific, Wilmington, USA) and digital images were acquired using a Leica DM IRB fluorescence microscope and with Leica TCS SP8 confocal microscope. Nonspecific staining of cells was observed in control incubations in which the primary antibodies were omitted.

### Western blot analysis

Cell pellets were homogenized in lysis buffer (Tris‐HCl pH 7.4, 1% Triton X100, NaCl 150 mmol/L and EDTA 1 mmol/L) supplemented with a cocktail of protease inhibitors (1:100, Sigma‐Aldrich, Saint Louis, Missouri, USA). For Western blot quantification, 50 μg of protein were electrophoresed on 12% SDS‐PAGE gels and transferred to nitrocellulose membranes. After blocking with 5% non‐fat milk powder in Tris‐buffered saline with 0.05% Tween‐20 (TBST), membranes were incubated overnight at +4°C with the following primary antibodies: rabbit caspase‐3 cleaved (1:1000, Cell Signaling, Danvers, Massachusetts, USA), mouse anti‐Cx47 (1:200, Invitrogen, Waltham, MA, USA), mouse anti‐Cx43 (1:1000, Cell Signaling, Danvers, Massachusetts, USA), rabbit anti‐Cx40 (1:200, Invitrogen, Waltham, MA, USA), mouse anti‐Cx36 (1:200, Invitrogen, Waltham, MA, USA), mouse anti‐Cx32 (1:500, Novex), rabbit anti‐Cx30 (1:200, Invitrogen, Waltham, MA, USA) and rabbit β‐tubulin (1:1000, Cell Signaling, Danvers, Massachusetts, USA). After three washes in TBST, the membranes were incubated with antimouse (1:20000, Jackson, West Grove, PA, USA) and anti‐rabbit HRP‐conjugated (1:50000, Jackson, West Grove, PA, USA) secondary antibodies for 1 hr at RT. Proteins bands were visualized with premixed ready‐to‐use chemiluminescent HRP detection reagent (Millipore, Darmstadt, Germany) according to the manufacturer's instructions and captured with an Uvitec Cambridge Imaging System. The density of each band was quantified using ImageJ analysis software and normalized to β‐tubulin levels measured in the same membranes.

### Monitoring cell adherence and proliferation

Cell adherence and proliferation was monitored in real‐time using the xCELLigence system E‐Plate. Different cell numbers were tested, with the *optimum* cell density to found to be 2.5 × 10^4^ cells/cm^2^ (data not shown). Experiments were performed at different time‐points and with various percentages of media derived from OEC cultures mixed with fresh medium, with 100% of fresh medium employed as a control. The impedance value of each well was monitored by the xCELLigence system for a time of 24 hrs and expressed as a cell index (CI) value. Data for cell adherence were normalized at 16 hrs after plating. Normalized CI, representing a quantitative measure of cell number, was calculated by dividing CI at the time‐point into the CI at the normalization time‐point (time 0). The Rate of Cell Growth (RCG) was determined by calculating the slope of the line between starting point and ending point.

### Cellular viability

Cells were trypsinized and adjusted to a concentration of 2.5 × 10^4^ cells/cm^2^ seeded in 96‐well plates (Costar) and incubated for 16 hrs with basal growth medium. Then medium was replaced with basal growth medium in control cultures and with 50% basal growth medium mixed 1:1 with OEC‐CM. Normoxic cultures were placed in both normal‐oxygen and in 1% oxygen conditions. Cellular viability was evaluated at time 0, 3, 8 and 24 hrs. Tests were performed adding a solution of 3‐(4,5‐dimethylthiazol‐2‐yl)‐2,5‐diphenyltetrazolium bromide (MTT; Sigma‐Aldrich, Saint Louis, Missouri, USA) at concentration of 5 mg/ml and placed for 2.5 hrs in a CO_2_ incubator. Media were gently removed, MTT solvent (DMSO, Sigma‐Aldrich, Saint Louis, Missouri, USA) was added, and cells were agitated on an orbital shaker for 5 min at RT. The absorbance was measured using a Varioskan Flash spectrophotometer (Thermo Scientific, Wilmington, USA) at 550 nm. Results were expressed as the percentage MTT reduction of control cells. The experiment was performed three times with six replicates per condition each time. Data are shown via standard box‐and‐whiskers plots in which the central‐line represents the median, the upper‐ and lower bounds of the boxes are min and max value and points represent all values expressed as percentage of control, assumed as 100%.

### Statistical analysis

n‐way Anova has been performed to determine the existences of interaction between n independent variables (time and treatment for two way anova and time, treatment and oxygen levels for three way Anova) on a continuous dependent variable. Tukey honest significant difference (HSD) has been used as post hoc test when Anova test indicated statistically significant differences to identify specific changes in time‐points or treatment conditions. Statistical calculation has been performed using R software (R Foundation for Statistical Computing). Values are represented in graphs as mean ±  standard error of the mean (SEM). All calculation was performed using GraphPad Prism v7 software.

## Results

### OEC‐CM exerts a protective effect in an *in vitro* H/R injury model

**Figure 1:**
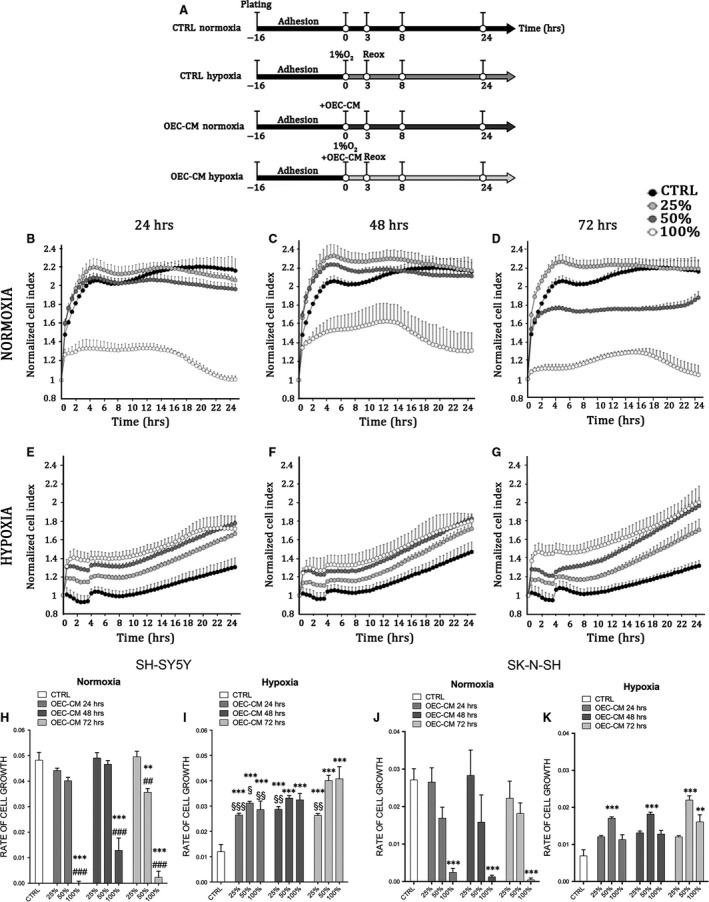
OEC‐CM exhibits neuroprotective effects on the human neuroblastoma cell lines. (**A**) Experimental plan. **(B**–**G)** xCELLigence system E‐Plate analysis of cell viability on SH‐SY5Y cell cultures. Each dot represents the CI mean (±SEM) of three independent analyses and CI was revealed every 30 min on the same cultures. (**H**–**K)** Rate of Cell Growth (slope of the line between time 0 and time 24) in normoxic and hypoxic SH‐SY5Y (**H**,** I**) and SK‐N‐SH (J,K) cell cultures exposed to different conditioning‐time and percentage of OEC‐CM.**P <* *0.01 *versus* CTRL. ***P < 0.001 *versus* CTRL. ##*P* < 0.01 *versus* corresponding OEC‐CM 25%. ###*P* < 0.001 *versus* corresponding OEC‐CM 25%. §*P* < 0.05 *versus* OEC‐CM 72 hr 100%. §§*P* < 0.01 *versus* OEC‐CM 72 hr 100%. §§§*P* < 0.001 *versus* OEC‐CM 72 hr 100%. (manovas and Tukey honest significant difference (HSD) post hoc test).

To induce hypoxia/reoxygenation (H/R) injury, 2.5 × 10^4^ cells/cm^2^ were plated in growth medium and incubated in a controlled humidified atmosphere at 37°C with constant 5% CO_2_ level. Sixteen hours after plating (time 0) hypoxic cultures were exposed to 3 hrs of hypoxia (1% O_2_) and then reoxygenated to 24 hrs. In OEC‐CM supplemented cell cultures the conditioned media was added at time 0 (Fig. 1A).

To investigate the potential protective effects of OEC‐CM on cell cultures after H/R injury, we supplemented growth medium with three different percentage of conditioned medium, 25%, 50% and 100%, collected from primary OECs cultures at different conditioning times (24, 48 and 72 hrs) (Fig. 1A). Our results showed that in normoxic conditions there was a significant reduction of normalized cell index (CI) in all cell cultures grown in 100% OEC‐CM (Fig. 1B–D), as result of reduced cell confluence and cell number. A similar result, although less marked than previous, was observed when the cells were cultured with 50% OEC‐CM collected after 72 hrs (Fig. 1D). This was probably linked to the lower level of serum and nutrients compared to the fresh medium. Cell cultures grown with 25% and 50% OEC‐CM, collected after 24 hrs and 48 hrs (Fig. 1A and B), did not show any significant difference compared to cells grown in 100% growth medium (CTRL) (Fig. 1A–C). Cells exposed to H/R injury, treated with 25%, 50% and 100% OEC‐CM collected at any time‐points, showed a significant increase of normalized CI compared to CTRL (Fig. 1E–G). Our findings also indicated that OEC‐CM, used at concentrations of 50% and 100%, exerted a higher protective effect on cells compared to 25% OEC‐CM and CTRL (Fig. 1E–G).

To further analyse, the effects of OEC‐CM on cell cultures, we evaluated the rate of cell growth (RCG) in both normoxic and H/R injured cultures (Fig. 1H–K). Our results demonstrated that in normoxic conditions, cell lines supplemented with 25% OEC‐CM, collected from OECs after 24, 48 and 72 hrs, did not show significant reduction of the RCG. The same effect was observed when cells were grown in 50% OEC‐CM collected after 24 or 48 hrs. In contrast, cultures grown in 50% OEC‐CM, collected after 72 hrs and in 100% of OEC‐CM (all conditioning times), showed a significant decrease of RCG (Fig. 1H and J). Interestingly, all conditioned media used at 25%, 50% and 100% on H/R injured cultures showed a significant increase of RCG compared to control cultures (Fig. 1I and K). Taken together, these findings suggested that, in both normoxic and H/R injured cultures, the cells grown in 48 hrs OEC‐CM mixed 1:1 in maintenance medium, showed a higher protective effect and supported a better RCG. For this reason all further studies have been performed by using 50% OEC‐CM collected after 48 hrs. These findings confirmed that OECs, affecting the media composition, were able to exert neuroprotective actions and to increase cell survival and cell proliferation after injury.

### OEC‐CM improves cell viability in H/R cultures

**Figure 2:**
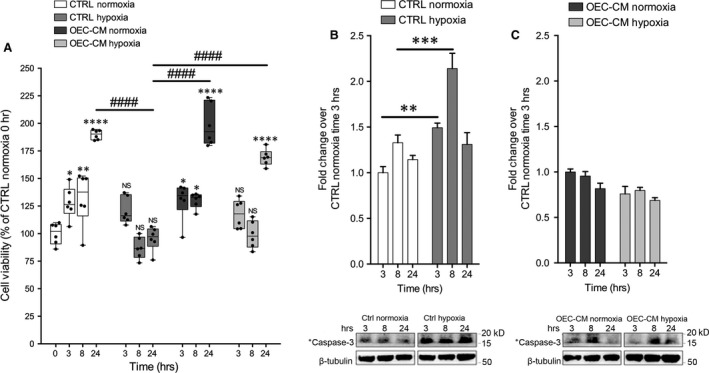
OEC‐CM increased SH‐SY5Y cell viability and reduced H/R injury mediated damage. (**A**) MTT‐viability tests performed at 0, 3, 8 and 24 hrs. Values of spectrophotometric determination at 550 nm are reported as percentage of cultures at time 0, considered as 100%. Data are shown via standard box‐and‐whiskers plots in which the central‐line represents the median, the upper‐ and lower bounds of the boxes are min and max value and dots represent all value expressed as percentage of control. NS not significant, *P < 0.05, **P < 0.01 and ***P < 0.001 *versus* CTRL Normoxia time 0. ####*P* < 0.0001 (manovas and Tukey honest significant difference (HSD) post hoc test). (**B**) Western blot analysis of Cleaved Caspase‐3 levels on SH‐SY5Y in control and H/R injured cultures. Proteins levels are plotted as fold change over 3 hrs after treatment in control culture considered as 1. **P < 0.01, ***P < 0.001 (manovas and Tukey honest significant difference (HSD) post hoc test). (**C**) Western blot analysis of Cleaved Caspase‐3 levels on SH‐SY5Y in OEC‐CM conditioned cultures in normoxic and in H/R injured cultures. Proteins levels are plotted as fold change over 3 hrs after treatment in OEC‐CM normoxic cultures considered as 1.

In order to investigate the effect of H/R condition on cell viability, we exposed hypoxic cell cultures to 1% O2 levels for 3 hrs and reoxygenation up to 24 hrs. Cell cultures viability was evaluated at different time‐points after injury (Fig. 2A).

Our results demonstrated that cells immediately after H/R injury and after 5 hrs of reoxygenation had significantly lower cell viability (MTT test). Furthermore, we established that the addition of OEC‐CM to the H/R injured cultures significantly improved cell viability compared to untreated cells. Otherwise cells cultured in presence of OEC‐CM for 24 hrs in normoxic conditions did not show any significant difference compared to control (Fig. 2A).

Further, we evaluated the levels of cleaved caspase‐3, a marker of apoptosis, in normoxic and H/R cultures with and without OEC‐CM. We found that cleaved caspase‐3 levels were significantly higher in hypoxic cultures, at all reoxygenation times, compared to normoxic cultures (Fig. 2B). Cells grown with OEC‐CM, in normoxia and exposed to H/R, did not show significant differences of cleaved caspase‐3 levels compared to unconditioned cultures (Fig. 2C).

### Expression levels of different Cxs in neuroblastoma cell lines under normoxic and hypoxic conditions

**Figure 3:**
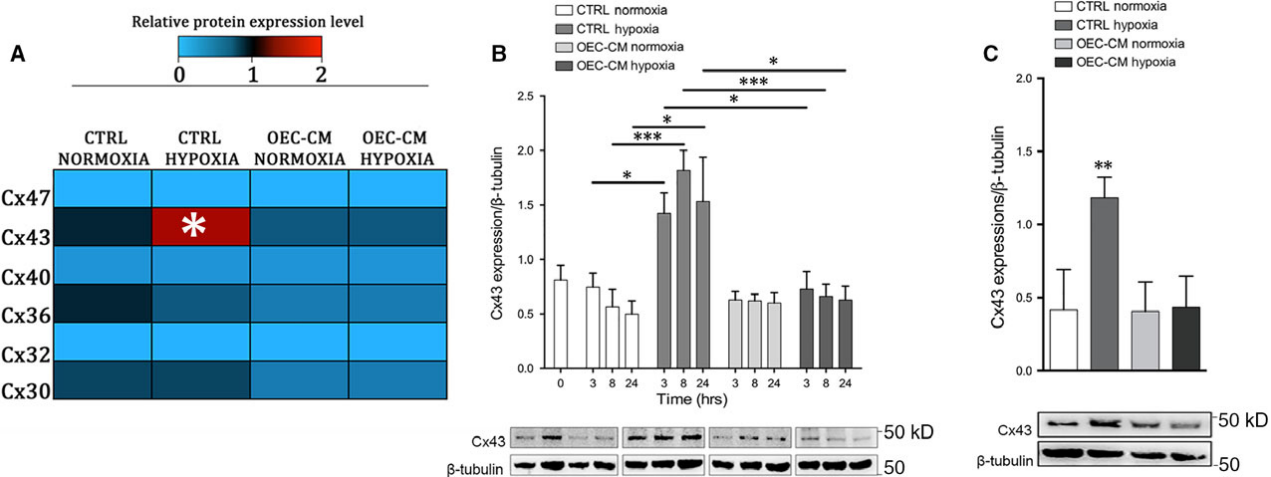
Cxs profile of SH‐SY5Y and SK‐N‐SH cell lines. (**A**) Heatmap of Cx47, Cx43, Cx40, Cx36, Cx32 and Cx30 protein expression levels on SH‐SY5Y cell line at 24 hrs. Western blots were digitally analysed by integrating the density of each protein band and its corresponding ß‐Tubulin band intensity. Colour key shown for each protein reveals the colour code used to visualise the relative protein expression level, light blue colours correspond to low relative protein expression levels, while red colour correspond to high relative protein expression levels. Average protein expression levels from duplicate cultures were assessed at 24 hrs post‐H/R injury. *P < 0.05 *versus* CTRL Normoxia. (manovas and Tukey honest significant difference (HSD) post hoc test). (**B**) Western blot analysis of Cx43 in lysates of SH‐SY5Y normoxic and H/R cultures. Data show the ratio between intensity of Cx43 bands divided by relative ß‐Tubulin bands intensity quantified using imageJ software. Blot shown is representative of three independent experiments. *P < 0,05, ***P < 0.001. (manovas and Tukey honest significant difference (HSD) post hoc test). (**C**) Western blot analysis of Cx43 in lysates of SK‐N‐SH normoxic and H/R cultures. Data show the ratio between intensity of Cx43 bands divided by relative ß‐Tubulin bands intensity quantified using imageJ software. Blot shown is representative of three independent experiments. **P < 0.01. (manovas and Tukey honest significant difference (HSD) post hoc test).

To identify if SH‐SY5Y cells, cultured in presence or in absence of OEC‐CM in normoxic and H/R conditions, expressed some specific Cxs (including Cx47, Cx43, Cx40, Cx36, Cx32, and Cx30), we performed Western blot analyses. Our data indicated that cells grown in all experimental conditions showed no or a very low expression of Cx47, Cx32, Cx40 and Cx30 (Fig. 3A). The cultures, maintained under normoxic conditions, showed basal expression levels for Cx36 and Cx43 that were reduced in presence of OEC‐CM. H/R injured cultures showed an intensely increased Cx43 levels and OEC‐CM was able to reduce this overexpression to normoxic levels (Fig. 3A).

**Figure 4:**
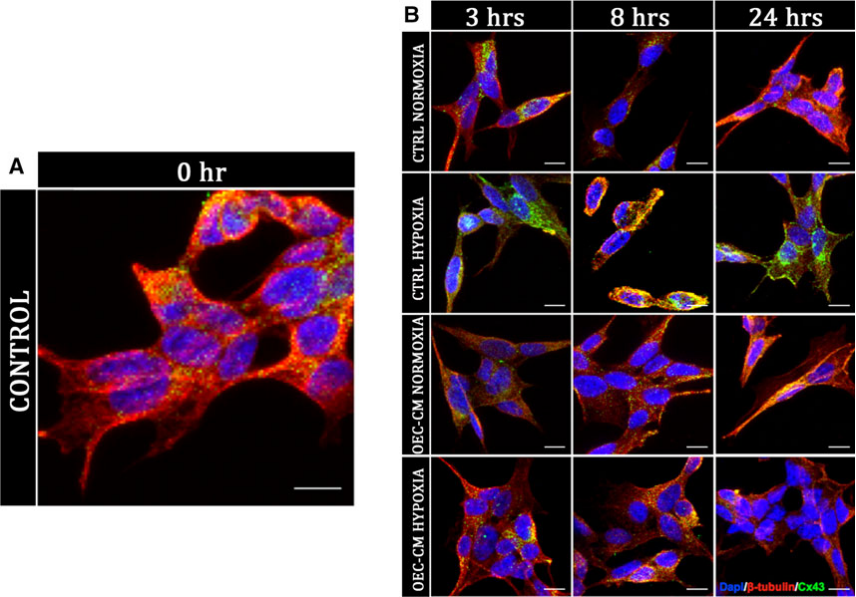
Cx43 immunofluorescence analysis on SH‐SY5Y cell cultures. Cx43 localization analysis by immunofluorescence on unconditioned control cultures (**A)** and on OEC‐CM treated cultures in normoxic condition and H/R injured cultures (**B**). Scale bar: 10 μm.

To better evaluate levels and localization of Cx43 in SH‐SY5Y cells we performed Western blots and immunofluorescence analysis at different time‐points. These experiments confirmed high levels of this marker from the end of the injury (3 hrs) in untreated SH‐SY5Y cells (Figs. 3B and 4A, B). The expression levels appeared to increase markedly after 8 hrs and then clearly decrease at 24 hrs (Fig. 3B) in both intracellular and extracellular compartments (Fig. 4B). However, our data showed that H/R cultures, in absence of OEC‐CM, had higher levels of Cx43 at each analysed time‐point compared to the cultures in normoxia with and without OEC‐CM. On the contrary, in injured cultures, in the presence of OEC‐CM the expression levels of Cx43 seemed unaffected along the different time‐points (Figs. 3A and B, 4B) compared to the cultures in normoxia with and without OEC‐CM. Moreover, H/R cultures exposed to OEC‐CM exhibited an evident reduction of Cx43 expression when compared to the unconditioned injured cultures (Figs. 3B and 4B).

To further confirm the increased Cx43 expression levels in hypoxic condition we performed Western blots analysis on SK‐N‐SH at 24 hrs (Fig. 3C). These experiments confirmed that also in this cell line the Cx43 levels were up‐regulated in H/R injured cultures while did not show any significant differences in OEC‐CM cultures compared to control cultures.

### Gap junction and/or hemichannel chemical inhibitors protect SH‐SY5Y cells from H/R injury

**Figure 5:**
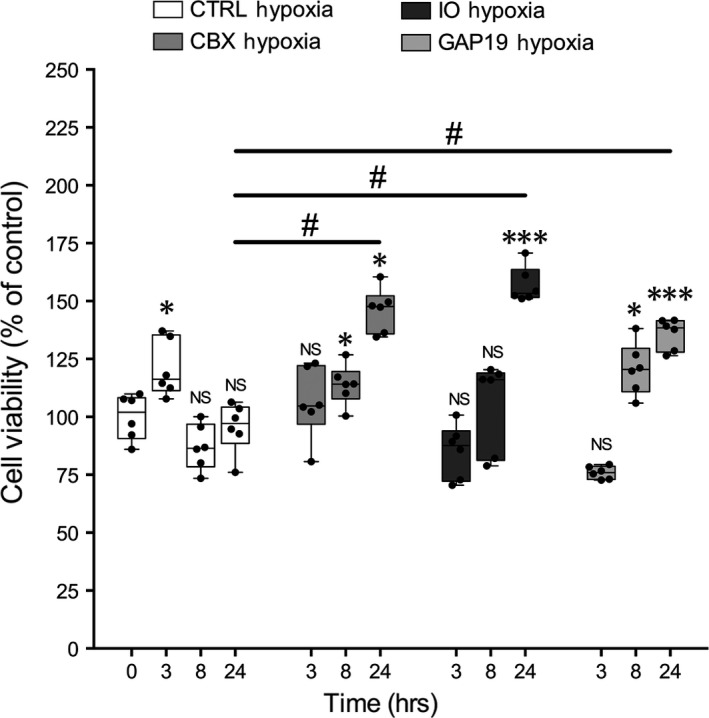
MTT‐viability tests on SH‐SY5Y exposed to H/R injury treated with a non‐selective GJIC inhibitor (CBX), selective inhibitor of homotypic Cx43‐GJs (IO) and, selective inhibitor of homotypic Cx43‐HCs (GAP19). MTT‐viability tests performed at 0, 3, 8 and 24 hrs. Values of spectrophotometric determination at 550 nm are reported as percentage of cultures at time 0, considered as 100%. Data are shown via standard box‐and‐whiskers plots in which the central‐line represents the median, the upper and lower bounds of the box are min and max value and dots represent all value. NS not significant, *P < 0.05 and ***P < 0.001 *versus* CTRL Normoxia time 0. #*P* < 0.05. (manovas and Tukey honest significant difference (HSD) post hoc test).

To analyse whether the neuroprotective effect on SH‐SY5Y cells exposed to H/R injury was related to GJ and/or HC functions of Cx43 we performed MTT‐viability test after adding GJ and/or HC inhibitors. We used CBX, a non‐selective GJIC inhibitor, IO to selectively target Cx43 homotypic GJs, and Gap19 to selectively inhibit Cx43 homotypic HCs. Results obtained from the titration of GJ and/or HC inhibitors (data not shown) suggested that the best inhibitor concentrations were 10 μM in culture medium. Our results demonstrated that H/R injured cultures treated with both GJ and HC chemical inhibitors significantly increase the cell viability over time compared to the control cultures exposed to H/R injury (Fig. 5).

## Discussion

The strengthening of some endogenous neuroprotective mechanisms as a mean by which to prevent and/or slow down the outcome of the various forms of neurodegenerative disorders has recently emerged as one of the most popular topics in applied neurobiology. It has been described that OECs exhibit the ability to promote regeneration in the damaged CNS, thus they could be involved as possible mediators of repair in neurological diseases 12, 14. As a source of different trophic factors, OECs have attracted an increasing interest as tool for regenerative medicine with applications that include spinal cord injury 55, 56 or axonal growth 57, 58 with a view towards new therapeutic approaches. It has been also demonstrated *in vitro* that the addition of OEC‐CM to the neuroblastoma SH‐SY5Y and SK‐N‐SH cells exposed to the neurotoxin 6‐hydroxydopamine (6‐OHDA) provides neuroprotective properties 59.

In this work we showed that OEC‐CM exerts a concentration‐ and time‐dependent protective effect on SH‐SY5Y and SK‐N‐SH cells subjected to H/R injury. Our aim was to investigate the molecular mechanism underlying this effect. A number of evidence revealed that, in the CNS, intercellular communication among neurons and glial cells via GJs and/or HCs could be critical in the spread of protective and/or deleterious signals. Cxs are dynamically expressed during injury and stress conditions and, for each condition and context, up‐ or down‐regulation of such proteins likely influencing gate properties of GJs and free HCs, may influence cell survival or cell death 21, 43, 44, 45, 46. In particular, several independent studies have pointed out that onset and progression of homeostatic imbalances observed during neurodegeneration could be associated with an enhanced HC activity in the CNS 60, 61, 62, 63, 64, 65, 66. Here we demonstrated *in vitro* that SH‐SY5Y and SK‐N‐SH cells grown in normoxia displayed no or low expression of Cx47, Cx43, Cx40, Cx36, Cx32 and Cx30 with or without the addition of OEC‐CM. When cells are cultured under hypoxic conditions, the Cx43 exhibited an increased expression whereas, the addition of OEC‐CM to the growth medium, restored the basal expression observed in normoxia. These evidence suggest that, while Cx43 may be involved in hypoxic response, the protective effect of OEC‐CM may be exerted through the modulation of this specific Cxs.

Cx43 is the principal astrocytic GJ protein in the CNS where it contributes to the formation of the functional syncytium, implicated in maintaining the homeostasis of the extracellular milieu of neurons 67, 68. Many studies support the potential therapeutic effects of Cx43‐GJ blockade on neuronal survival in various models of injury including stroke, epilepsy, ischaemia, optic nerve damage and spinal cord injury, with GJ communication and HC opening leading to increased secondary damage via the inflammatory response 69, 70, 71.

To investigate the possible interplay between OEC‐CM and Cx43 in protection after H/R injury, the effects of Cx43 chemical inhibition has been assessed. When H/R stress is induced in SH‐SY5Y cells, both Cx43‐GJ and Cx43‐HC chemical inhibition significantly increased the cell viability over time compared to control cultures. The functional modulation of Cx43 provides additional support on its involvement in OEC‐CM mediated neuroprotection, likely exerted through the prevention of the spread of injury signals. One appealing hypothesis is that OEC‐CM works by influencing Cx43 expression in the SH‐SY5Y cells via paracrine factors likely involved in the physiological role of OECs within the CNS.

It is noteworthy that several studies have demonstrated a role of Cx proteins in the regulation of tissue homeostasis occurring independently of their channel activities, in the context of cell growth, adhesion, migration, apoptosis and signalling 72.

While further investigations are needed to unveil the molecular details of such neuroprotection, these data point to the possibility that the proposed model may be useful in the context of therapeutic applications after brain injury. The involvement of Cxs in maintaining the delicate balance of CNS cells, via GJs and/or HCs, may indeed stimulate the development of new modulators for Cxs‐based channels as novel therapeutic agents for the cure of nervous disorders 73, 74, 75, 76.

## Conflicts of Interest

The authors confirm that there are no conflicts of interest.

## Author contributions

N.V. and R.Pa. designed the research study; N.V., G.C. and R.Pe. performed experiments; N.V. and S.F. collected and analysed data; C.P. and L.V. provided some reagents and instruments; A.G., A.Z., C.P. and V.C. gave technical support and conceptual advice; N.V., G.C. and R.Pa. wrote the manuscript.
